# Association of low attenuation area scores with pulmonary function and clinical prognosis in patients with chronic obstructive pulmonary disease

**DOI:** 10.1515/biol-2022-0871

**Published:** 2024-08-14

**Authors:** Xiangli Tang, Chentao Xu, Tianjin Zhou, Yanfei Qiang, Yingzhe Wu

**Affiliations:** Department of Radiology, Changxing People’s Hospital, Huzhou 313100, Zhejiang, China; Department of Respiratory, Changxing People’s Hospital, Huzhou 313100, Zhejiang, China

**Keywords:** COPD, LAA score, pulmonary function, clinical prognosis, correlation study

## Abstract

The objective of this study was to investigate the relationship between low attenuation area (LAA) scores, pulmonary function parameters, and clinical prognosis in patients with chronic obstructive pulmonary disease (COPD). COPD patients were divided into four LAA-based grades. Various lung function parameters were measured and correlated with LAA scores. Patient symptoms were examined using the St. George’s Respiratory Questionnaire (SGRQ) and exercise capacity using the 6-min walk test (6MWT). Statistical analysis determined the significance of differences. Higher levels of LAA were associated with decreased lung function and airflow limitations, suggesting a positive relationship between the two. Clinical symptom scores increased as COPD severity based on LAA stratification worsened. Reduced exercise capacity was shown by a substantial decline in 6MWT scores as COPD severity increased. As LAA scores increased, SGRQ scores increased, indicating a decreased quality of life (QOL). The study demonstrated a relationship between LAA scores and COPD severity. High LAA scores were associated with poor lung function, worse clinical symptoms, limited exercise capacity, and lower QOL. These findings show that LAA scores are clinically relevant for disease severity assessment and COPD management. Further research is required to determine LAA scores’ prognostic significance in disease progression and treatment response to enhance COPD therapy.

## 1 Introduction

Chronic obstructive pulmonary disease (COPD) is a prevalent as well as progressive respiratory disorder identified by persistent airflow limitation and inflammation of the airways. [1]. The disease is primarily characterized by irreversible and progressive airflow limitation, airway wall inflammation, and reduced lung elasticity due to frequent exposure to harmful gases or particles. The condition presents with a variety of clinical phenotypes and symptoms including shortness of breath, chronic cough, and chest tightness, which dramatically impact patients’ quality of life (QOL) and exercise capacity. It is a major global health concern primarily caused by long-term exposure to harmful inhalants, such as cigarette smoke and air pollution. COPD imposes a great burden on individuals, families, and healthcare systems due to its high morbidity and mortality rates. In fact, it ranks as the third highest cause of mortality globally [1,2] and resulted in 3.2 million deaths globally in 2015 [2]. Furthermore, the number of COPD cases is predicted to double by 2030. As individuals with COPD age and the disease progresses, the economic burden on patients increases, and their QOL declines. Consequently, it is crucial to explore new diagnostic and treatment approaches for COPD [3].

Moreover, the heterogeneity and complexity of COPD make its assessment and management challenging. The current evaluation of COPD severity primarily relies on lung function tests, symptom questionnaires, history of acute exacerbations, and the Global Initiative for Chronic Obstructive Lung Disease (GOLD) guidelines. Nonetheless, these traditional methods often fail to fully capture the multifaceted nature of the disease and its impact on patients’ lives. Moreover, COPD is a dynamic disease with varying symptoms and severity levels, which can further complicate its management.

The assessment of COPD severity and prognosis plays a critical role in guiding clinical decision-making and optimizing patient care [2,3]. Various parameters, including pulmonary function (PF) tests and clinical assessments, are used to evaluate the functional status and prognosis of individuals with COPD [4]. However, these methods have limitations in accurately reflecting the heterogeneity of COPD pathology and manifestations. One such parameter is the low attenuation area (LAA) score, derived from computed tomography (CT) imaging, which provides a quantitative measure of emphysematous changes in the lungs [4,5]. It has been demonstrated that COPD patients exhibit heterogeneity, and a single PF test has limitations in assessing the disease. High-resolution CT (HRCT) allows for the direct assessment of small airway lesions and ductal wall thickening, providing a more accurate reflection of the changes in lung histopathology in COPD patients. However, the role of imaging phenotypes in the comprehensive evaluation of COPD severity remains unclear [5].

The LAA score, commonly determined using automated analysis software, has been shown to correlate with the extent of emphysema and disease severity in COPD patients [5,6]. Emphysema, characterized by the destruction of lung tissue, impairs gas exchange and reduces PF [7]. Previous studies [8] have reported a relationship between LAA scores and lung function parameters such as forced expiratory volume in one second (FEV1) and forced vital capacity (FVC). However, the association between PF, LAA scores, and clinical prognosis in COPD patients remains an area of ongoing research [9]. The LAA score is the primary method for evaluating respiratory tract and alveolar lesions in HRCT scans [10,11]. Specifically, in chest HRCT scans of COPD patients, it refers to the area of low-density lesion region that appears due to emphysema [12]. The numerical size of the LAA score visually reflects the size of the lesion area [13,14]. The relationship between the LAA score and comprehensive evaluation indices for COPD patients is not clear [15]. However, the association between PF, LAA scores, and clinical prognosis in COPD patients remains an area of ongoing research. Therefore, this study aims to reveal the correlation between LAA scores, PF, as well as clinical prognosis in COPD patients, providing a basis for the selection of COPD treatment plans.

## 2 Materials and methods

### 2.1 General data

**Figure 1:**
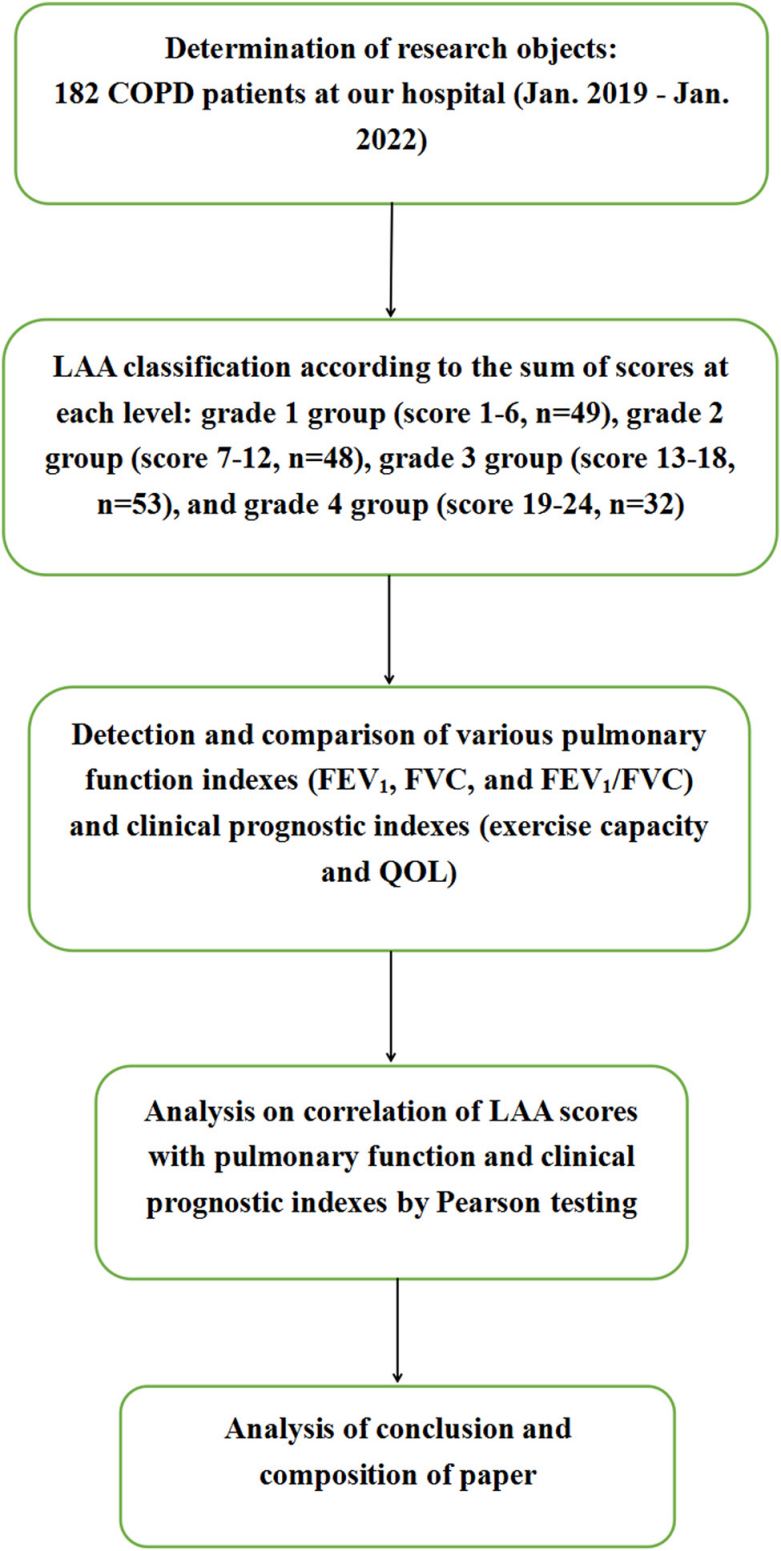
Technology roadmap.


A total of 182 patients with COPD who were hospitalized in our hospital from January 2019 to January 2022 were included in this observational study using a cross-sectional design, including 108 males and 74 females, with an age range of 43–76 years and a mean age of 58.73 ± 10.09. The duration of the disease ranged from 1 to 9 years, with a mean duration of 5.03 ± 2.67. According to the severity of COPD, the patients were divided into the following four groups: 49 cases of Grade 1 group, 48 cases of Grade 2 group, 53 cases of Grade 3 group, and 32 cases of Grade 4 group. There was no statistical difference between the four groups. Statistical analysis of the collected data was conducted using SPSS 26.0. Ethical approval for the study was obtained from the hospital’s ethics committee, and the study adhered to the principles delineated in the 2013 Declaration of Helsinki [16]. Prior to participation in the study, the researchers provided the research subjects with a comprehensive explanation of the study’s purpose, significance, potential benefits, and risks. Informed consent was obtained, with subjects or their family members signing the consent form when the subjects were unable to provide a signature. The technology roadmap is shown in Figure 1.

**Informed consent:** Informed consent has been obtained from all individuals included in this study.
**Ethical approval:** The research related to human use has been complied with all the relevant national regulations, institutional policies and in accordance with the tenets of the Helsinki Declaration, and has been approved by the Changxing People’s Hospital Ethics Committee.

### 2.2 Inclusion and exclusion criteria

The study involved patients who met the diagnostic criteria for COPD as specified in the GOLD guidelines [17]. Inclusion criteria for participants were as follows: a post-bronchodilator FEV1/FVC ratio < 0.70, arterial partial pressure of oxygen (PaO_2_) < 8 kPa (60 mmHg), and/or arterial partial pressure of carbon dioxide (PaCO_2_) > 6.67 kPa (50 mmHg) while breathing room air, along with evident clinical symptoms of COPD. Additionally, patients were required to be in a clinically stable stage, without experiencing exacerbations within the past 4 weeks, and have complete imaging data available.

Exclusion criteria included patients with malignant tumors, immune system disorders, or those currently undergoing immunotherapy, as these conditions may confound the interpretation of the study results. Furthermore, individuals who were pregnant or lactating were also excluded from the study due to potential risks and ethical considerations.

### 2.3 HRCT scan

The patients underwent an HRCT scan using the Philips Ingenuity 64-slice spiral CT scanner. Prior to scanning, respiratory training was conducted to ensure optimal conditions. Scans were taken with patients lying supine, head first, and arms naturally raised, encompassing the entire lung field from the thoracic inlet to the diaphragm level at maximum exhalation. Additionally, a magnifying scan of the upper lobe of the right lung (from the lung apex to 5 cm below the tracheal carina) was performed at maximum inhalation. The scan parameters were as follows: effective tube voltage of 120 kV, tube current of 100 mAs, matrix of 512 × 512, pitch of 0.195, collimation of 128 × 0.625 mm, rotation time of 0.5 s, layer spacing of 5 mm, layer thickness of 5 mm, FOV of 35 × 35 cm, scanning speed of 146.4 mm/s, and image reconstruction with a 1 mm layer thickness.

LAA scores, which indicate the extent of emphysematous changes in the lungs, were measured using CT scans [18]. The CT scans were performed using a standardized protocol, with slice thickness and reconstruction settings optimized for lung imaging. LAA scores were calculated using computer software that automatically quantified the percentage of lung voxels with attenuation values below a specified threshold.

After the HRCT scan, the obtained images were processed using the COPD analysis software on an image analysis workstation. This software provided three-dimensional reconstruction images of the lungs and displayed emphysematous tissue smaller than the set threshold in red. Emphysematous areas were defined as CT values < −950 HU and were quantified as the LAA in each selected lung field level. Manual scoring was conducted by radiologists at three anatomical levels: 1 cm above the upper edge of the aortic arch, 1 cm below the carina level, and 3 cm above the right septum, representing the upper, middle, and lower lung field levels, respectively. The corresponding LAA score was assigned based on the area percentage: LAA < 5% – score of 0; 5% ≤ LAA < 25% – score of 1; 25% ≤ LAA < 50% – score of 2; 50% ≤ LAA < 75% – score of 3; and LAA ≥ 75% – score of 4.

LAA scores were further classified based on the sum of scores at each level: total score of 0 – Grade 0; total score of 1–6 – Grade 1; total score of 7–12 – Grade 2; total score of 13–18 – Grade 3; and total score of 19–24 – Grade 4. To ensure quality control, the emphysema measurement was independently performed by two radiologists with more than 2 years of experience, who were blinded to the patients’ medical history. In case of significant discrepancies in total scores, the two radiologists reassessed and reached a consensus on a score.

### 2.4 PF

Lung function parameters were measured using spirometry. Participants were instructed to perform maximal forced expiration into the spirometer, following standardized guidelines [19]. These parameters included FEV1, FVC, bronchial wall thickness (WT) (measured in millimeters), bronchial wall thickening (BWT) (measured in millimeters), maximal inspiratory pressure, maximal expiratory pressure, and emphysema index (EI) expressed as a percentage.

The Cosmed Quark PFT series PF instrument was used to conduct routine pulmonary ventilation function tests and bronchodilation tests. These tests included the measurement of FEV1, FVC, and the FEV1/FVC ratio.

To evaluate the exercise capacity of patients, the 6-min walk test (6MWT) [20] was performed. This test is a commonly used submaximal exercise testing method that assesses physical fitness by measuring the distance covered in a defined period of time.

The CAT score was utilized to reveal the impact of COPD on patient health. It comprises eight items that focus on symptoms such as chest tightness, cough, sputum production, and breathlessness. The scoring scale for each item ranges from 0 to 5, leading to a total score that can vary between 0 and 40. Scores within the range of 0–10 indicate mild symptoms, 11–20 indicate moderate symptoms, 21–30 indicate severe symptoms, and 31–40 indicate very severe symptoms.

To evaluate the QOL of patients, the St. George’s Respiratory Questionnaire (SGRQ) [17] was employed. This questionnaire consists of 50 items and 76 questions that explore respiratory symptoms, activity limitations, and the overall impact of the disease on daily life. A higher total score, out of 100, indicates poorer QOL.

### 2.5 Clinical symptoms

Clinical symptoms, such as cough, sputum production, chest tightness, exercise limitation, impact on daily activities, emotional well-being, sleep quality, and energy levels, were assessed using a questionnaire [21]. The questionnaires utilized a scoring system ranging from 0 to 10 to quantify the severity of these symptoms, providing a comprehensive evaluation of their degree of impact.

### 2.6 Statistical disposal

The data were analyzed with the aid of SPSS 26.0 statistical software. Measurement data were presented as mean ± standard deviation (mean ± SD). One-way analysis of variance was conducted for comparison, and pairwise comparisons were verified by the *t*-test. Count data were expressed as rates (%). Comparison of count data was performed using the *χ* ^2^ test, and pairwise comparisons were verified using the Spearman correlation analysis. Statistical significance was set at *p* < 0.05.

## 3 Results

### 3.1 Comparison of PF indexes

**Figure 2:**
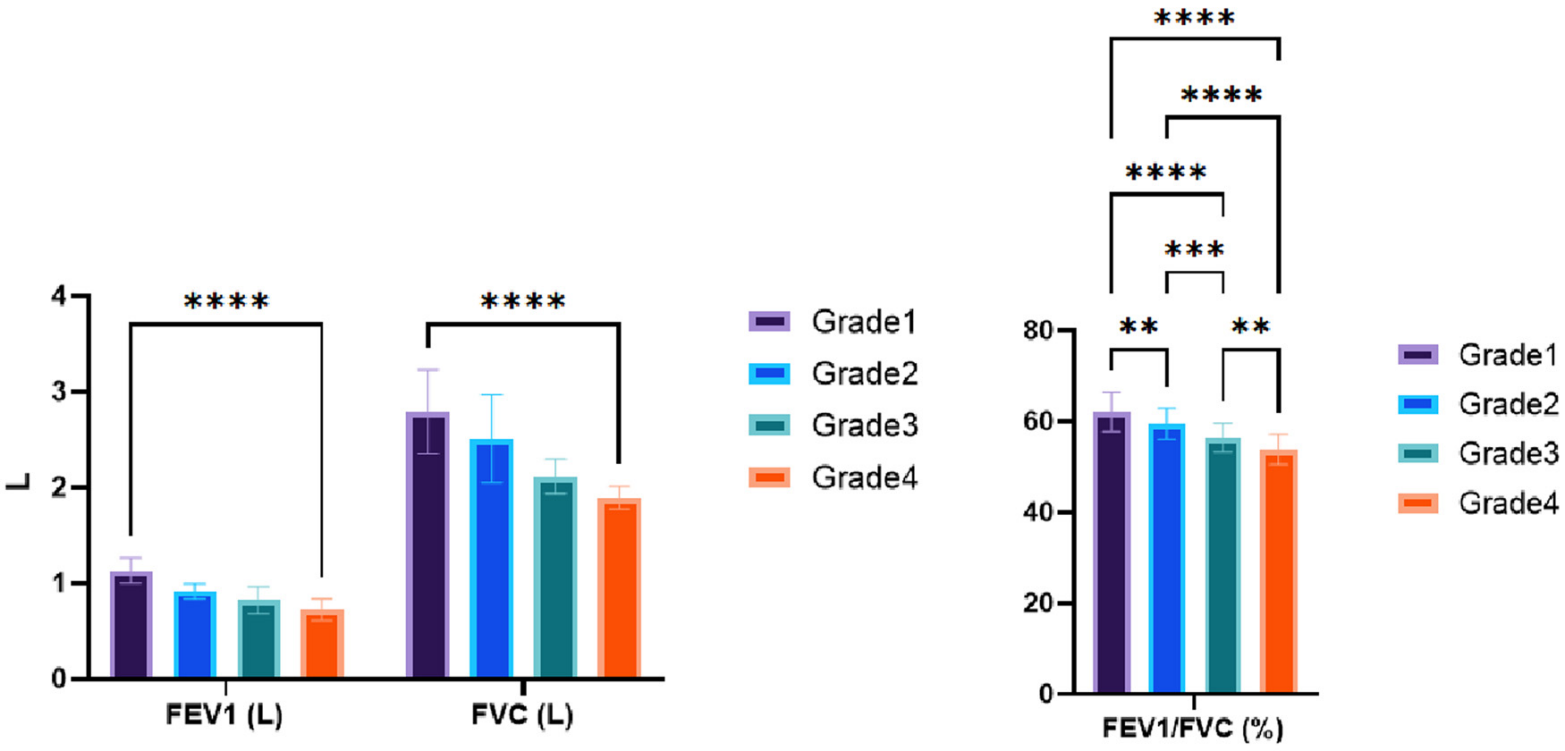
The comparison of PF indexes among different grades of COPD patients with LAA stratification.

This study aimed to discover the relationship between different grades of COPD patients, categorized based on LAA scores and PF indexes. A total of 182 COPD patients were included in the study. The results revealed a significant decline in PF as the COPD grade increased. Specifically, the Grade 4 group exhibited the most severe impairment in lung function, as indicated by lower mean values for FEV1, FVC, and FEV1/FVC ratio. Statistical analysis confirmed significant differences in PF indexes among the different COPD grades (*p* < 0.001 for all comparisons). These findings emphasize the significance of assessing LAA scores in evaluating COPD severity and determining appropriate treatment strategies. Further research is warranted to explore the clinical implications and prognosis associated with these findings (Table 1 and Figure 2).

**Table 1:** Comparison of PF indexes among different grades of COPD patients with LAA stratification (mean ± standard deviation)

Groups	*N*	FEV_1_ (L)	FVC (L)	FEV_1_/FVC (%)
Grade 1 group	49	1.13 ± 0.13	2.79 ± 0.44	62.04 ± 4.30
Grade 2 group	48	0.91 ± 0.08	2.51 ± 0.46	59.46 ± 3.34
Grade 3 group	53	0.82 ± 0.14	2.11 ± 0.18	56.49 ± 3.17
Grade 4 group	32	0.72 ± 0.11	1.89 ± 0.12	53.81 ± 3.34
*F*		96.024	57.528	40.842
*p*		<0.001	<0.001	<0.001

### 3.2 Comparison of lung structural measurements and EI

**Figure 3:**
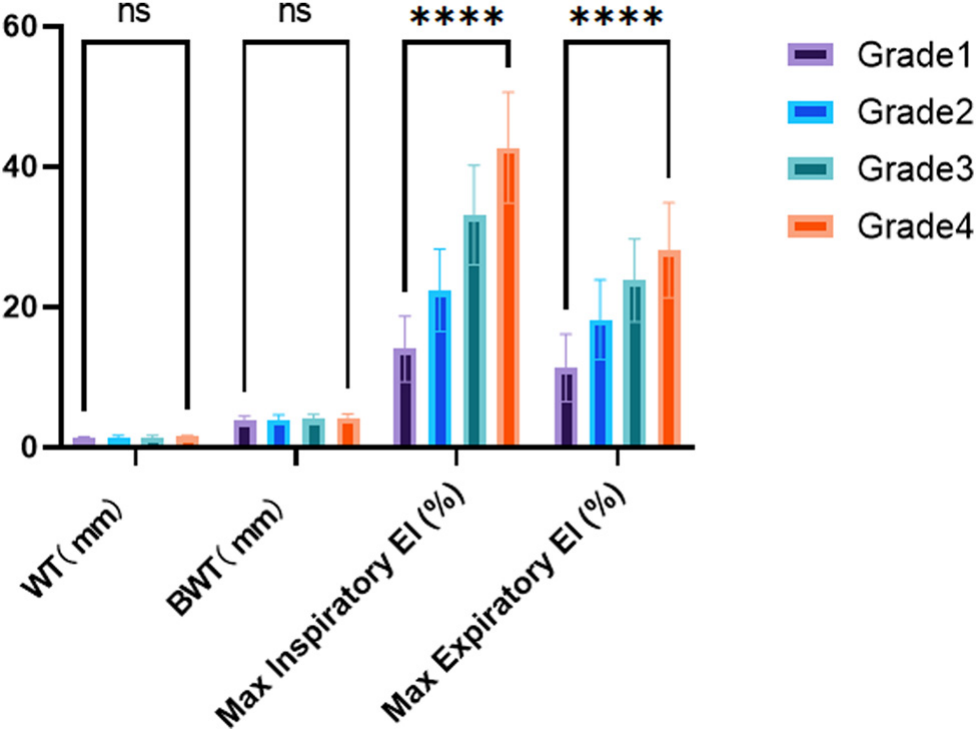
Comparison of lung structural measurements and EI among different grades of COPD patients with LAA stratification.

To evaluate lung structural measurements and EI in different grades of COPD patients with LAA stratification, Table 2 presents the mean values and standard deviations of WT, BWT, maximal inspiratory EI, and maximal expiratory EI for each grade of COPD patients. The results demonstrated a significant increase in WT, BWT, maximal inspiratory EI, and maximal expiratory EI as the COPD grade increased. Statistical analysis confirmed significant differences in these measurements between each grade of COPD patients (*p* < 0.0001 for all comparisons). These findings suggest a progressive increase in lung structural abnormalities and emphysema severity as COPD severity, based on LAA stratification, increases (Figure 3).

**Table 2:** Comparison of lung structural measurements and EI among different grades of COPD patients with LAA stratification (mean ± standard deviation)

Groups	WT (mm)	BWT (mm)	Max inspiratory EI (%)	Max expiratory EI (%)
Grade 1 group	1.34 ± 0.19	3.82 ± 0.61	14.0 ± 4.7	11.3 ± 4.8
Grade 2 group	1.40 ± 0.27	3.98 ± 0.63	22.4 ± 5.9	18.2 ± 5.7
Grade 3 group	1.44 ± 0.27	4.11 ± 0.55	33.1 ± 7.1	23.8 ± 5.9
Grade 4 group	1.51 ± 0.19	4.23 ± 0.54	42.7 ± 7.9	28.1 ± 6.8
*p* (1 vs 2)	0.9994	0.9896	<0.0001	<0.0001
*p* (1 vs 3)	0.9971	0.9384	<0.0001	<0.0001
*p* (1 vs 4)	0.9910	0.8921	<0.0001	<0.0001
*p* (2 vs 3)	0.9998	0.9939	<0.0001	<0.0001
*p* (2 vs 4)	0.9976	0.9728	<0.0001	<0.0001
*p* (3 vs 4)	0.9993	0.9967	<0.0001	<0.0001

### 3.3 Comparison of clinical symptoms among different grades of COPD

**Figure 4:**
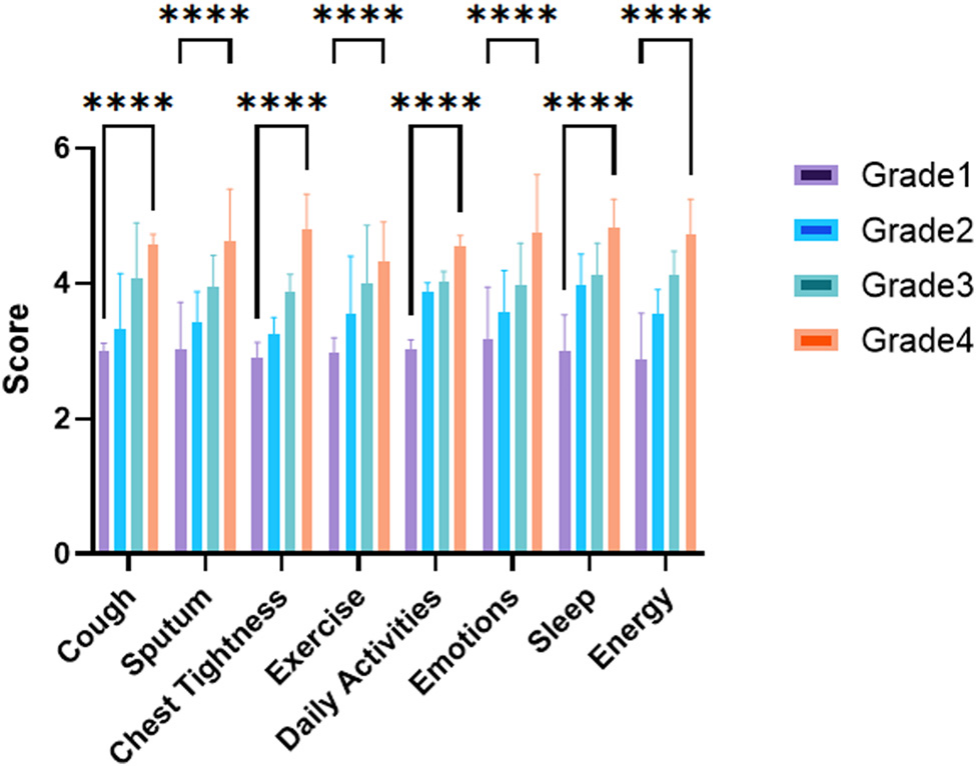
Comparison of clinical symptoms among different grades of COPD patients with LAA stratification.

To compare clinical symptoms among different grades of COPD patients with LAA stratification, Table 3 displays the mean values and standard deviations of cough, sputum production, chest tightness, exercise limitation, impact on daily activities, emotional well-being, sleep disturbances, and energy levels for each grade of COPD patients. As the COPD grade increased, there was an overall trend toward higher symptom scores across all categories. However, statistical analysis showed no significant differences in symptom scores between grade 1 and grade 2 (*p* > 0.05), as well as between grade 1 and grade 3 (*p* > 0.05). Nevertheless, a trend towards higher symptom scores in Grade 4 compared to Grade 1 was observed, with chest tightness showing statistically significant differences (*p* = 0.0635). These findings suggest that as COPD severity, based on LAA stratification, increases, there is a worsening of clinical symptoms overall. Further research is warranted to explore the impact of these symptoms on patients’ QOL and to develop targeted interventions for symptom management in different grades of COPD patients (Figure 4).

**Table 3:** Comparison of clinical symptoms among different grades of COPD patients with LAA stratification

	Cough	Sputum	Chest tightness	Exercise	Daily activities	Emotions	Sleep	Energy
Grade 1	3.00 ± 0.11	3.01 ± 0.71	2.89 ± 0.23	2.97 ± 0.22	3.02 ± 0.14	3.17 ± 0.77	2.99 ± 0.54	2.87 ± 0.68
Grade 2	3.33 ± 0.81	3.41 ± 0.46	3.24 ± 0.25	3.55 ± 0.85	3.87 ± 0.14	3.57 ± 0.62	3.96 ± 0.47	3.54 ± 0.36
Grade 3	4.08 ± 0.81	3.95 ± 0.46	3.88 ± 0.25	4.01 ± 0.85	4.03 ± 0.14	3.97 ± 0.62	4.12 ± 0.47	4.11 ± 0.36
Grade 4	4.57 ± 0.15	4.62 ± 0.77	4.81 ± 0.51	4.33 ± 0.57	4.55 ± 0.15	4.76 ± 0.85	4.82 ± 0.42	4.73 ± 0.51
*p* (1 vs 2)	0.9169	0.8630	0.9040	0.6698	0.3460	0.8636	0.2312	0.5590
*p* (1 vs 3)	0.1349	0.2368	0.1962	0.1599	0.1813	0.3790	0.1083	0.0635
*p* (1 vs 4)	0.0318*	0.0259*	0.0046*	0.0429	0.0383*	0.0287*	0.0079*	0.0066*
*p* (2 vs 3)	0.4439	0.7054	0.5801	0.7966	0.9889	0.8562	0.9890	0.6684
*p* (2 vs 4)	0.1377	0.1530	0.0326*	0.5282	0.6379	0.1645	0.4420	0.1651
*p* (3 vs 4)	0.8222	0.6358	0.3527	0.9422	0.7925	0.5006	0.6014	0.6924

* indicate statistical significance (*P < 0.05).

### 3.4 Comparison of scores of PF, exercise capacity, and QOL

**Figure 5:**
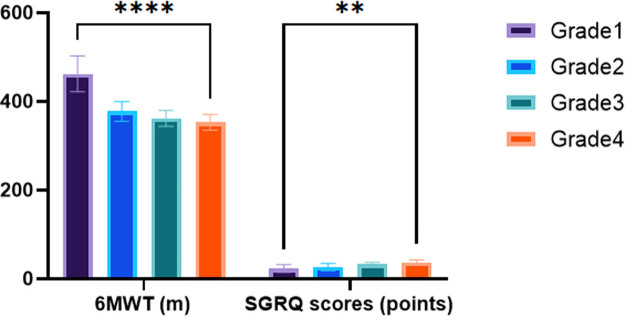
Comparison of scores of exercise capacity and QOL.

Table 4 presents the mean values and standard deviations of the 6MWT and SGRQ scores for each grade of COPD patients. Results indicated a significant decrease in the 6MWT scores, suggesting declining exercise capacity, as the COPD grade increased (*F* = 161.572, *p* < 0.001). Additionally, a progressive increase in the SGRQ scores was observed, indicating a poorer QOL with increasing COPD severity (*F* = 34.788, *p* < 0.001). Statistical analysis confirmed significant differences in both the 6MWT and SGRQ scores among the different COPD grades (*p* < 0.001 for both comparisons). These findings indicate a significant impairment in exercise capacity and a substantial negative impact on patients’ QOL as COPD severity, based on LAA stratification, increases (Figure 5).

**Table 4:** Comparison of scores of exercise capacity and QOL (*x* ± *s*)

Groups	6MWT (m)	SGRQ scores (points)
Grade 1 group	462.39 ± 40.46	23.24 ± 8.29
Grade 2 group	377.50 ± 22.34	26.71 ± 8.25
Grade 3 group	361.79 ± 17.92	33.11 ± 4.22
Grade 4 group	352.97 ± 17.74	37.22 ± 5.43
*F*	161.572	34.788
*p*	<0.001	<0.001

### 3.5 Correlation of LAA scores with various indexes

**Figure 6:**
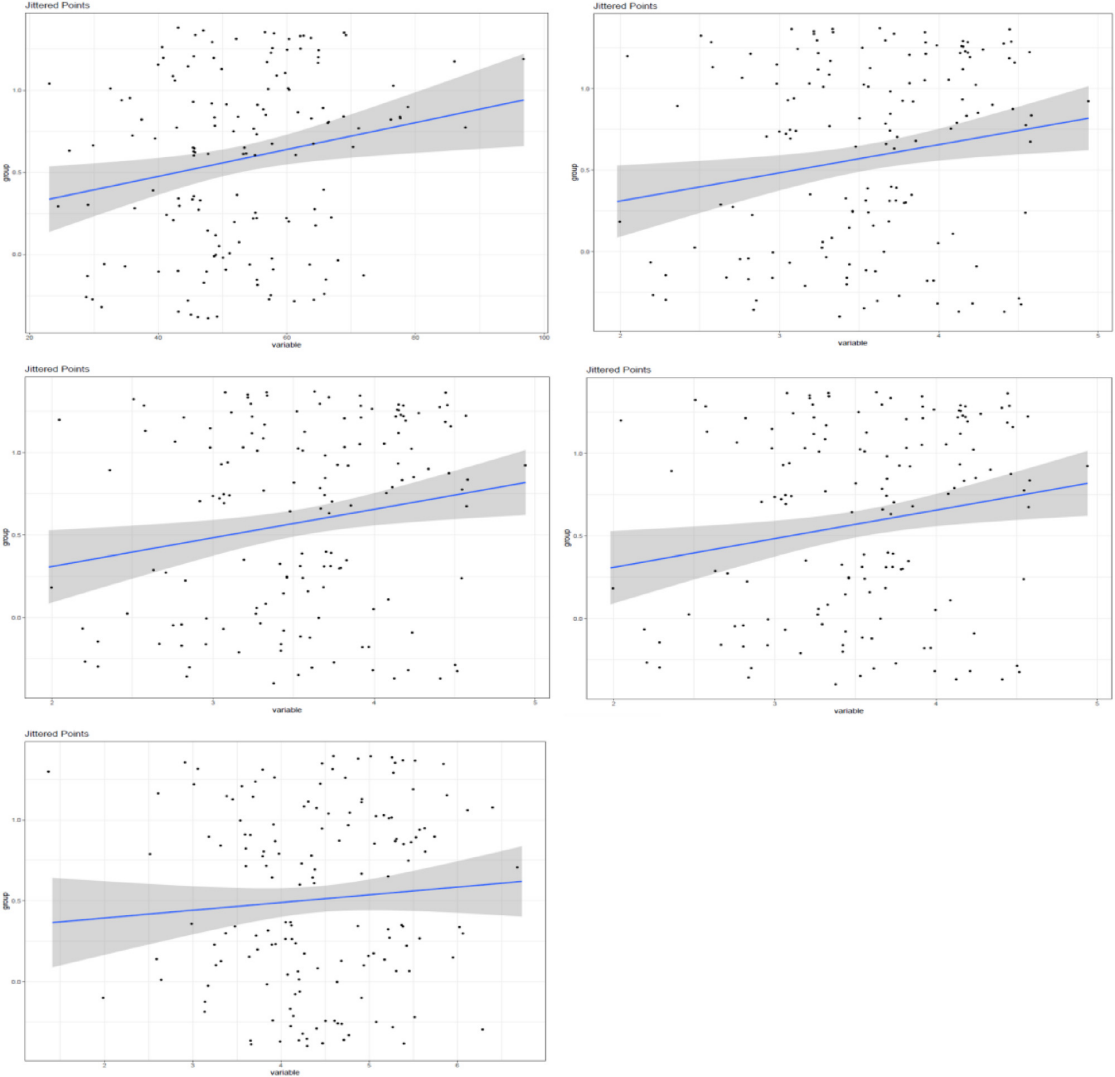
Analysis of the correlation of LAA scores with various indexes.

To analyze the correlation between LAA scores and various indexes in patients with COPD, correlation analysis was performed (Table 5). The LAA scores exhibited a strong positive correlation with FEV1 (*r* = 0.858, *p* < 0.001), FVC (*r* = 0.855, *p* < 0.001), and FEV1/FVC ratio (*r* = 0.828, *p* < 0.001). These findings indicate that as LAA scores increase, there is a significant decrease in lung function, as evidenced by lower FEV1 and FVC values, along with an increased likelihood of airflow limitation based on the FEV1/FVC ratio. The LAA scores also demonstrated a moderate positive correlation with the 6MWT distance (*r* = 0.653, *p* < 0.001), suggesting that higher LAA scores are associated with reduced exercise capacity in COPD patients. Furthermore, a strong positive correlation was observed between LAA scores and higher SGRQ scores (*r* = 0.811, *p* < 0.001), implying that increased LAA scores are associated with a lower QOL. These results highlight the clinical significance of LAA scores as a marker of disease severity and their impact on lung function, exercise capacity, and QOL in patients with COPD (Figure 6).

**Table 5:** Analysis of correlation of LAA scores with various indexes

Indexes	*r*	*p*
FEV_1_	0.858	<0.001
FVC	0.855	<0.001
FEV_1_/FVC	0.828	<0.001
6MWT	0.653	<0.001
SGRQ scores	0.811	<0.001

## 4 Discussion

COPD is a prevalent and significant respiratory disease [20], serving as the leading cause of death globally, with a continuously increasing fatality rate [22]. Acute exacerbations are responsible for the majority of COPD-related deaths, with an 8% fatality rate during hospitalization, rising to 23% within 1 year of follow-up [23,24]. The development of COPD is closely associated with the occurrence of airway or alveolar lesions, which stem from frequent exposure to noxious gases or suspended particles [15,16]. These noxious stimuli trigger a cascade of pathological processes, resulting in irreversible airway narrowing, airway wall inflammation, and emphysema caused by reduced lung elasticity. These changes in the anatomical structure of COPD manifest as irreversible airflow limitation and reduced ventilation [17,20]. COPD is a heterogeneous syndrome with complex pathogenesis and various clinical phenotypes, and the current evaluation of COPD patients primarily relies on the GOLD guidelines as a means for clinicians to assess their condition [25,26].

The main objective of this study was to investigate the relationship between EI, specifically the LAA scores, and various clinical parameters in patients diagnosed with COPD. According to the severity of COPD, the patients were divided into four groups. There were no statistically significant differences in age and gender between the four groups. However, it should be noted that our study population may not be representative of all COPD patients, as it was limited to a specific geographic location or patient group, thereby limiting the generalizability of the study findings. Further research is necessary to address these limitations and enhance the overall validity of the results. Our findings provide valuable insights into how COPD severity impacts clinical symptoms, exercise capacity, and QOL.

Our analysis revealed a strong positive correlation between LAA scores and lung function parameters, such as the FEV1, FVC, and FEV1/FVC ratio. Thus, as LAA scores increase, lung function significantly decreases, indicating a worsening of airflow limitation. These results support the use of LAA scores as a reliable marker for assessing the severity of COPD.

Furthermore, our study demonstrated that as COPD severity increased based on LAA stratification, there was a noticeable trend toward higher symptom scores in all categories, indicating a deteriorating clinical symptomatology. Although statistical analysis did not reveal significant differences in symptom scores between Grade 1 and Grade 2 or between Grade 1 and Grade 3, there was an evident trend toward higher symptom scores in Grade 4 compared to Grade 1, with statistical significance observed for chest tightness. These findings suggest an overall aggravation of clinical symptoms as COPD severity progresses, particularly in more advanced stages of the disease.

Additionally, our results demonstrated a significant decrease in exercise capacity, as measured by the 6MWT scores, as the severity of COPD increased based on LAA stratification. This means that higher LAA scores are strongly associated with reduced exercise capacity in COPD patients. Moreover, the SGRQ scores exhibited a progressive increase with increasing COPD severity, indicating a decline in the patients’ QOL. These findings underscore the considerable negative impact of COPD severity as determined by LAA scores on the exercise capacity and overall QOL of patients.

The robust positive correlations observed between LAA scores and various clinical parameters reaffirm the clinical significance of LAA scores as an indicator of disease severity in COPD patients. Therefore, LAA scores can be utilized by healthcare professionals to personalize treatment plans for COPD patients. Patients with higher LAA scores indicating more extensive lung damage may require more aggressive treatment strategies, such as targeted pharmacotherapy, pulmonary rehabilitation, or referral for lung transplantation. By considering LAA scores alongside other clinical parameters, healthcare professionals can tailor treatment plans to the specific needs of each individual. The findings suggest that higher LAA scores are linked to poorer lung function, reduced exercise capacity, and lower QOL. These results highlight the importance of considering LAA scores in the assessment and management of COPD patients.

Zhu et al. [27] found that the heterogeneous phenotype of COPD requires a combination of multiple evaluation methods. The diagnostic efficacy of combining LAA, visual subtypes, and basic characteristics achieves good consistency with current diagnostic criteria. Chen et al. [28] found that a lower value of the DSP was related to a greater worsening of symptoms, more frequent exacerbations, poorer PF, and more severe emphysema (higher LAA%). These readily determined parameters, including the DSP and LAA%, can serve as indicators for assessing the COPD clinical course and may serve as a guide to corresponding treatments. Our study affirms the clinical significance of the LAA score as an indicator of disease severity in patients with COPD, a finding that fills a gap in past research. In addition to the significant correlations observed, our study has several strengths.Initially, we conducted a comprehensive assessment of multiple clinical parameters, including lung function, exercise capacity, and QOL, providing a holistic understanding of the impact of COPD severity on these domains. This comprehensive approach enhances the clinical relevance and applicability of our findings. Moreover, our study employed a robust statistical analysis, strengthening the validity and reliability of our results. By performing rigorous statistical analyses, we have ensured the accuracy and robustness of the observed associations. These strengths underscore the significance and reliability of our study’s findings.

However, it is important to acknowledge certain limitations in our study. First, the study population was limited to a specific geographical location or a particular patient group, which may limit the generalizability of the findings. To address this limitation, we need future studies with more diverse populations to validate our results in different settings. Second, due to the cross-sectional nature of our study, it is not possible to establish a cause-and-effect relationship between the evaluated clinical parameters and LAA scores. Therefore, further longitudinal studies are necessary to explore the predictive value of LAA scores in disease progression and treatment response.

In conclusion, our study highlights a strong association between LAA scores and the severity of COPD in patients. Higher LAA scores were strongly correlated with poorer lung function, worsening clinical symptoms, reduced exercise capacity, and a lower QOL. These findings emphasize the clinical relevance of LAA scores as an important tool for assessing disease severity and guiding the management of COPD patients. Further research is warranted to investigate the predictive value of LAA scores and their utility in informing treatment decisions, thereby enhancing the understanding and care of patients with COPD.
